# Au Nanoparticle Synthesis in the Presence of Thiolated Hyaluronic Acid

**DOI:** 10.3390/ijms262110532

**Published:** 2025-10-29

**Authors:** Lyudmila V. Parfenova, Eliza I. Alibaeva, Guzel U. Gil’fanova, Zulfiya R. Galimshina, Ekaterina S. Mescheryakova, Leonard M. Khalilov, Semen N. Sergeev, Nikita V. Penkov, Challapalli Subrahmanyam

**Affiliations:** 1 Institute of Petrochemistry and Catalysis, Ufa Federal Research Center, Russian Academy of Sciences, Prospekt Oktyabrya, 141, 450075 Ufa, Russia; 2 Department of Materials Science and Physics of Metals, Institute of Technology and Materials, Ufa University of Science and Technology, 12 Karl Marx Street, 450008 Ufa, Russia; 3 Institute of Cell Biophysics of the Russian Academy of Sciences, Federal Research Center “Pushchino Scientific Center for Biological Research of the Russian Academy of Sciences”, Institutskaya 3, 142290 Pushchino, Russia; 4 Department of Chemistry, Indian Institute of Technology Hyderabad, Kandi, Sangareddy 502284, Telangana, India

**Keywords:** au nanoparticles, thiolated hyaluronic acid, scanning transmission electron microscopy, photon cross-correlation spectroscopy, X-ray photoelectron spectroscopy

## Abstract

Gold nanoparticles (AuNPs) are of significant interest due to their unique properties and applications in biomedicine. While hyaluronic acid (HA) has been used to modify pre-formed AuNPs, its thiolated derivative (HA−SH) has been less explored for the direct synthesis and stabilization of AuNPs. This study investigates the use of thiolated hyaluronic acid as a key component in the synthesis of AuNPs. A series of HA-AuNPs (**HA-AuNP1-4**) were synthesized by reacting HA-SH with HAuCl_4_ at different mass ratios. The resulting nanoparticles were characterized using UV-Vis spectroscopy, scanning/transmission electron microscopy (SEM/STEM), X-ray photoelectron spectroscopy (XPS), photon cross-correlation spectroscopy (PCCS), and zeta potential measurements. The chemical transformations of the thiol ligand were studied using NMR spectroscopy. The morphologies and sizes of AuNPs depended on the HA-SH-to-HAuCl_4_ ratio, ranging from icosahedral and triangular particles (≥146 nm) to quasi-spherical particles with a bimodal distribution (6–7 nm and 45–60 nm). XPS confirmed the presence of metallic gold (Au^0^) and a Au−S bond, while NMR and XPS revealed the partial oxidation of thiol groups to sulfonic acid. Zeta potential measurements showed that lower HAuCl_4_ concentrations resulted in higher negative charge (up to −41.5 mV), enhancing colloidal stability. This work demonstrates a versatile approach to the synthesis of hyaluronic acid-based gold nanomaterials with tunable properties for potential biomedical applications.

## 1. Introduction

In recent decades, gold nanoparticles (AuNPs) have become the subject of a rapidly and exponentially growing number of studies. Due to their numerous beneficial properties, gold nanoparticles have secured a strong position in the global market. Gold nanoparticles are produced in a variety of forms and are characterized by a large surface area, high electrical conductivity, bioinertness, stability, and excellent solubility. AuNPs are used in fields such as catalysis, optics, sensors, delivery of therapeutic agents, photodynamic therapy, and electronics [1,2,3,4,5].

Thus, the unique properties and high demand for AuNPs determine significant interest in these materials in scientific and practical fields. Fine-tuning the physical and chemical parameters of metal nanoparticles is possible due to advances in synthesis methods, which allow for variation in their composition, size, morphology, and stability.

The following three main reasons for the success of AuNPs are noted: (1) high chemical and physical stability, which also ensures biocompatibility, (2) ease of surface functionalization with organic and biological molecules, and (3) a variety of optical properties related to the presence of the surface plasmon resonance effect [6,7].

There are many methods for synthesizing AuNPs, which can be divided into two main classes: “top-down” or “bottom-up” [8]. “Bottom-up” transformations are of great interest because, by varying the nature of the precursor, reducing agent, stabilizer, and reaction conditions, it becomes possible to control the physical and chemical properties of the resulting nanoparticles. AuNPs are predominantly synthesized through the chemical reduction of Au(III) salts, utilizing key methodologies that differ in their stabilizing agents and the properties of the resulting nanoparticles. A cornerstone method, developed by Turkevich et al., employs citrate ions in aqueous solution to reduce HAuCl_4_, where citrate acts as a dual-function reducing and stabilizing agent [9,10]. However, the citrate–gold bond is relatively weak, allowing for subsequent ligand exchange. In contrast, the Brust–Schiffrin method represents a major advancement for producing exceptionally stable AuNPs. This two-phase synthesis uses sodium borohydride (NaBH_4_) as a strong reducing agent and thiol ligands as stabilizers [11]. The resulting thiol-capped AuNPs, characterized by a strong covalent gold–sulfur bond, exhibit superior stability and low polydispersity and can be easily functionalized for further applications [4]. Our previous work has shown that AuNP synthesis can be performed using organoaluminum reagents as reductants in organic solvents, with subsequent hydrolysis in the presence of a tertiary thiol [12]. An eco-friendly synthesis of AuNPs using renewable resources, such as plant extracts, microorganisms, and biomolecules (peptides, polysaccharides), is known as well [13,14]. This method utilizes non-toxic reagents and is characterized by its relative simplicity. However, it often results in a broader distribution of particle sizes and shapes, as well as lower reproducibility.

The literature contains a substantial number of studies on the synthesis or use of AuNPs in the presence of various polysaccharides or the decoration of nanoparticles with polysaccharides [15,16,17,18]. Modification of inorganic components with polysaccharide‒hyaluronic acid is used to develop materials employed in disease diagnostics, theranostics, drug delivery, and gene therapy [19]. Hyaluronic acid can be utilized as a reducing agent, as well as a stabilizing, non-fouling (antifouling), and biomolecule-targeting (biotargeting) ligand for nanoparticles [19]. For example, gold nanoparticles decorated with hyaluronic acid (HA) can serve as CD44-targeted anticancer agents [20]. AuNPs and thiol-modified biomacromonomers derived from hyaluronic acid (HA) and gelatin have been applied to form printable semisynthetic extracellular matrix (sECM) hydrogels [21]. The synthesis of AuNPs is achieved by the reaction of HAuCl_4_ with hyaluronic acid (HA) [22] or in the presence of HA [23,24,25,26]. Dicarboxylated hyaluronate (DCH) and cellulose (DCC) with controlled composition and molecular weight have been used as reducing and stabilizing agents in an environmentally friendly one-step synthesis of AuNPs [27]. Thiolated hyaluronic acid has only been utilized as a modifier of pre-formed gold nanoparticles (AuNPs) [20,21,28,29]. The introduction of thiol groups is intended to promote the formation of strong Au^0^–S interactions, thereby enhancing nanoparticle stability.

The aim of this study is to explore the potential use of thiolated hyaluronic acid for the simultaneous synthesis and stabilization of gold nanoparticles. The study investigated the transformation of HA-SH in reaction with HAuCl_4_ and the morphology, composition, size, and charge of the resulting gold particles.

## 2. Results and Discussion

### 2.1. HA-AuNP Synthesis

**Scheme 1:**
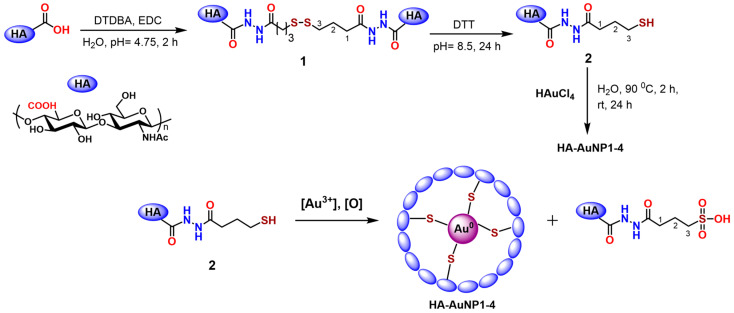
Synthesis of **HA-AuNP1-4**.

To obtain AuNPs, in the first stage, the synthesis of –S–S– and –SH derivatives of hyaluronic acid **1** and **2**, respectively, was carried out according to the method described in Refs. [30,31]. Using the polysaccharide dithiodihydrazide and the water-soluble dehydrating agent 1-ethyl-3-[3-(dimethylamino)propyl]carbodiimide (EDC) in a ratio of 2:1:1.5, the HA conjugate **1** was obtained with a substitution degree of approximately 40%. To obtain the HA derivative with terminal SH groups, the reducing agent DTT (dithiothreitol, Cleland’s reagent) was added to the reaction mixture (Scheme 1). Compounds **1** (HA–S–S–HA) and **2** (HA–SH) were purified by dialysis. Next, compound **2** was reacted with HAuCl_4_ at a mass ratio of 1:(1–0.1) in bidistilled water and then heated to 90 °C for 2 h. Then, the mixture was left to stir at room temperature for 24 h, resulting in a series of HA-AuNPs: [HA]:[HAuCl_4_] = 1:0.25 (**HA-AuNP1**), 1:0.225 (**HA-AuNP2**), 1:0.20 (**HA-AuNP3**), and 1:0.175 (**HA-AuNP4**).

**Figure 1:**
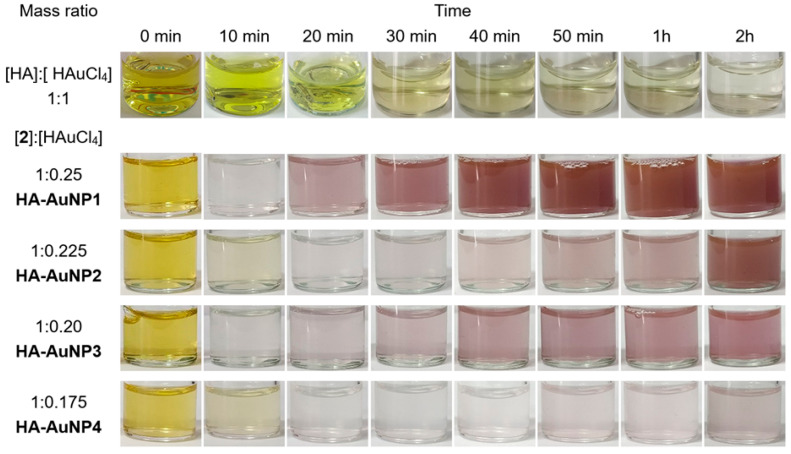
The color of solutions depending on time and the ratio of reagents **HA-AuNP1-4**.

As shown in Figure 1, the color of the AuNP solutions gradually changed from yellow to violet-brown during the reaction and from light violet to brown with increasing concentration of HAuCl_4_. The reaction carried out in the presence of hyaluronic acid at a 1:1 mass ratio for 2 h did not lead to the visible formation of AuNPs.

### 2.2. HA-AuNP Characterization

#### 2.2.1. UV–Visible Spectroscopy

**Figure 2:**
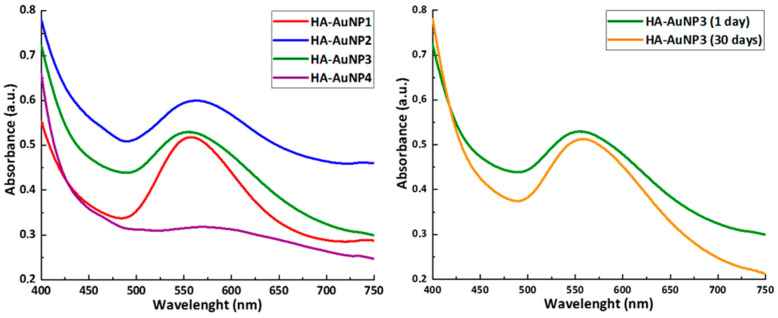
UV-Vis spectra of HA-AuNPs.

The UV–visible absorption spectra of the **HA-AuNP1** to **HA-AuNP4** samples, shown in Figure 2, exhibited peaks in the range of 550–570 nm (Table 1). This is characteristic of the surface plasmon resonance (SPR) of gold nanoparticles (AuNPs), which depends on their size, shape, and aggregation state [32,33,34,35]. The presence of a small peak in the UV spectrum for **AuNP4**, along with the pale violet color of the solution, may indicate a low concentration of particles, which is reflected in the decreased intensity.

For samples **AuNP2** and **AuNP3**, the absorption maximum around 450 nm reached a higher absorbance value than that of **AuNP1**, despite their surface plasmon resonance peak (SPR) being broader and less defined. The primary reason for this is likely the presence of a number of small particles (nuclei) and/or gold ions in the colloidal solution. A lower concentration of HAuCl_4_ was used in the synthesis of **AuNP2** and **AuNP3**. This likely led to a slower reaction rate and incomplete reduction of the gold ions. While some of the ions formed stable nanoparticles (which produce the broad SPR peak), a significant portion remained as very small, unstable nuclei. The less distinct SPR peak suggests a polymodal distribution of particle sizes and shapes. All these findings warrant further, more detailed investigation into the reduction kinetics using UV-Vis and XPS.

We conducted a comprehensive stability study using two methods: monitoring optical properties via UV-Vis spectroscopy and tracking the hydrodynamic particle size via PCCS. The measurements were performed for the samples after synthesis (1 day) and after 30 days of storage in an aqueous solution. The absorption spectra recorded at a 30-day interval are almost completely identical (Figure 2). The plasmon resonance peak, characteristic of AuNPs, retains its position in the 550–570 nm range. An increase in peak intensity is observed, which may be associated with the ripening of the particles. This is consistent with the data from PCCS (Figure S4) and indicates the relative stability of the particles.

The particle size distribution curves for day 1 (Figure S3) and day 30 (Figure S4) are practically identical. The average diameter remained unchanged for the smallest particles, 6–7 nm after 1 day and 6.5–7.5 nm after 30 days with a somewhat increased peak intensity. The size of particles in the 45–60 nm range shifted to the 70–90 nm region, which might be associated with the onset of aggregation. Nevertheless, the distribution remains narrow and bimodal, which is evidence of the relative stability of the obtained AuNPs.

Visual inspection revealed no signs of aggregation or sedimentation after 30 days: the colloidal solution remained transparent and retained its characteristic purple color without the formation of visible precipitate.

#### 2.2.2. Electron Microscopy and Photon Cross-Correlation Spectroscopy

For further investigation of the morphology and structure of the samples, both scanning electron microscopy (SEM) and bright-field scanning transmission electron microscopy (BF-STEM) were used. These studies demonstrated that the shape and size of the particles depend on the synthesis conditions.

**Figure 3:**
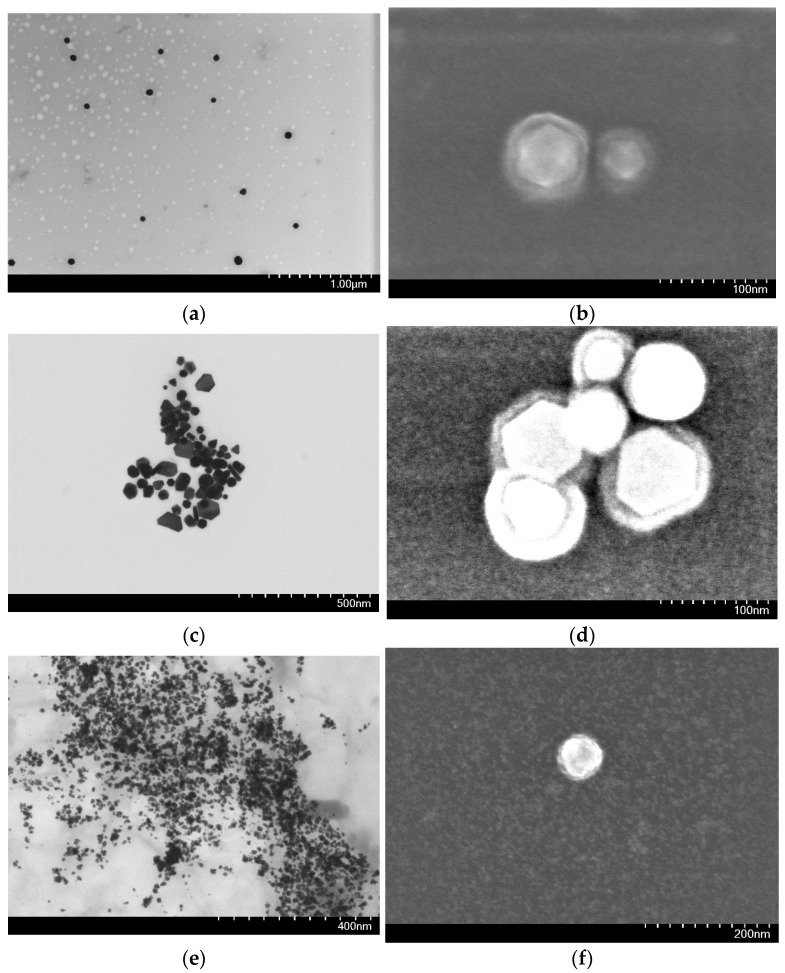
BF-STEM and SEM images of AuNPs; microscopy characterization of precipitates at ×35,000 ((**a**), **HA-AuNP1**), ×350,000 ((**b**), **HA-AuNP1**), ×90,000 ((**c**), **HA-AuNP2**), ×350,000 ((**d**), **HA-AuNP2**), ×130,000 ((**e**), **HA-AuNP3**), and ×200,000 ((**f**), **HA-AuNP3**) magnifications (corresponding micrometer and nanometer scale bars shown on each image).

Figure 3 presents micrographs of **HA-AuNP1**, **HA-AuNP2**, and **HA-AuNP3**. SEM analysis confirmed that the synthesized particles are within the nanometer range and exhibit different morphologies depending on the concentration of HAuCl_4_. The dark brown **HA-AuNP1** solution, as revealed by BF-STEM analysis, contains nanodisperse icosahedral particles measuring within the nanoscale (Figure 3b and Figure S5a–f), as well as equilateral triangular particles (Figure S5g–i) with sizes exceeding the nanometer range (diameter 146–172 nm). The particle size of **HA-AuNP1**, determined by PCCS, ranged from 65 to 90 nm (Figure S1). The icosahedral gold nanoparticles are surrounded by a modified hyaluronic acid shell with a thickness varying between 12 and 18 nm (Figure S5e).

In contrast, the **HA-AuNP2** solution consists exclusively of nanodisperse particles with heterogeneous shapes ranging from 40 to 70 nm (Figure 3c and Figure S6a–f). These AuNPs exhibit icosahedral, pentagonal, prismatic, trapezoidal, and triangular morphologies. Notably, hyaluronic acid forms a shell approximately 15 nm thick around smaller gold particles (~40 nm), while larger particles (60–70 nm) are coated with a shell measuring 15–20 nm in thickness (Figure S6d–f). PCCS size analysis showed particle diameters between 55 and 70 nm (Figure S2).

Unlike the previous two, the light violet **HA-AuNP3** solution contains only nanodisperse quasi-spherical particles enveloped by a polysaccharide shell, exhibiting a bimodal size distribution of 8–13 nm and around 50 nm (Figure 3e,f and Figure S7a–f). PCCS measurements also revealed a bimodal distribution for **HA-AuNP3** particles, with size populations centered at approximately 6–7 nm and 45–60 nm, respectively (Figure S3).

#### 2.2.3. X-Ray Photoelectron Spectroscopy

**Figure 4:**
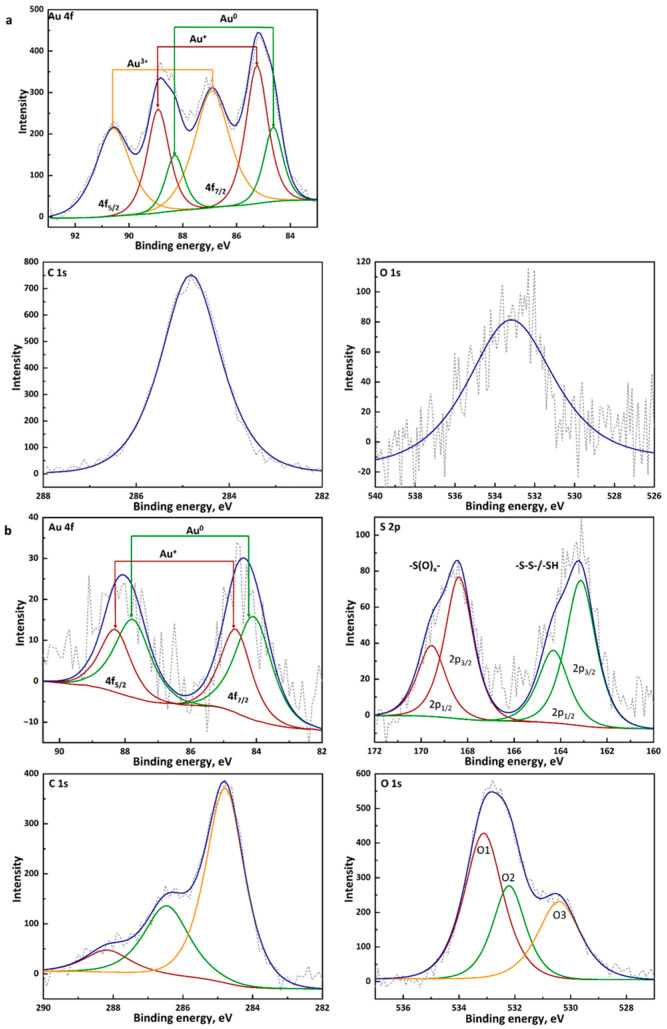
High-resolution XPS spectra for Au 4f, C 1s, and O 1s of HAuCl_4_ (**a**) and **HA-AuNP3** (**b**). The experimental data (dashed line) are fitted with individual components (solid lines in various colors).

To determine the degree of Au^3+^ conversion during the reaction, XPS analysis of the initial hydrogen tetrachloroaurate (HAuCl_4_) was performed (Figure 4a, Table 2 and Table S1). High-resolution spectra were obtained for the Au 4f, C 1s, and O 1s regions. All spectra were calibrated using the position of the C 1s peak from hydrocarbon contamination, set at 284.8 eV [36]. The spectrum in the Au 4f region (Figure 4a) exhibits a characteristic complex structure typical of gold(III) compounds. The spectrum was fitted with six components, grouped into three doublets with a fixed spin–orbit splitting of ~3.67 eV [37,38,39] and an area ratio of 4f_7_/_2_/4f_5_/_2_ = 4/3 [40]. The doublet with Au 4f_7_/_2_ binding energy of 86.9 eV is the main one and is characteristic of Au^3+^ ions [39,41,42]. This state is dominant in the sample.

The C 1s spectrum was fitted with one intense, symmetric peak at a binding energy of 284.8 eV, which corresponds to carbon from adsorbed hydrocarbons (C–C/C–H) used for spectrum calibration [36]. The absence of other components confirms that carbon is not part of the initial inorganic compound.

The O 1s spectrum is described by one broad peak centered at 533.2 eV. The presence of oxygen is likely associated with adsorbed water or the hydration of HAuCl_4_·xH_2_O crystals, which is typical for this reagent [43]. The peak position is consistent with the presence of oxygen in hydroxyl groups and/or adsorbed water [44].

During the XPS analysis, the results of which are shown in Figure 4b, it was demonstrated that the spectrum of the obtained **HA-AuNPs** (using **HA-AuNP3** as an example) is characterized by main peaks, 4f_7_/_2_ and Au 4f_5_/_2_, spin–orbit splitting values of about 3.67 eV, and a peak intensity ratio of 1.33, which is typical for gold [38,40,45,46]. The peak of Au at approximately 84.1 eV indicates the presence of Au^0^, which is typical for nanoparticles with a preserved metallic structure [38,40,45,46]. The presence of the Au 4f_7_/_2_ peak at a higher binding energy (84.6 eV) indicates the presence of an oxidized form of gold (Au^1+^) [47]. Comparison of the spectra of the initial HAuCl_4_ and **HA-AuNP3** shows that Au^3+^ is completely consumed during the reduction. The content of Au^0^ increased in the final sample **HA-AuNP3** (Table 2).

Oxygen O1s is represented as three components: O1 533.1 (47.0%), O2 532.2 (25.4%), and O3 530.4 (27.6%). The O1 peak at 532.1 eV can be attributed to the hydroxyl group [48,49]. The O2 component (532.2 eV) apparently corresponds to oxygen atoms of the amide bond, carboxylic group, or titanium hydroxide of the substrate. The O3 signal may belong to titanium oxide present on the metal substrate [50].

The XPS spectra contain two sulfur S2p signals at 168.9 eV and 163.7 eV. The peak at 163.7 eV corresponds to sulfide sulfur bound to the gold surface [51]. The peak at 168.9 eV may indicate a higher oxidation state of sulfur –SO_x_–, which may point to the oxidation of disulfide/thiol ligands [52,53,54,55]. The extent of thiol oxidation was assessed from the XPS spectra according to Ref. [56]. The quantitative ratio of –SH and –SO_x_– forms, established by determining the signal intensities, was approximately 1:1: the proportion of sulfur in the SH-form was 52.2% and in the oxidized form was 47.8%.

Thus, as follows from the XPS spectra, the thiol group in the polysaccharide was partially oxidized to a sulfoxide, whose structure was clarified by us using NMR spectroscopy.

#### 2.2.4. NMR Spectroscopy

**Figure 5:**
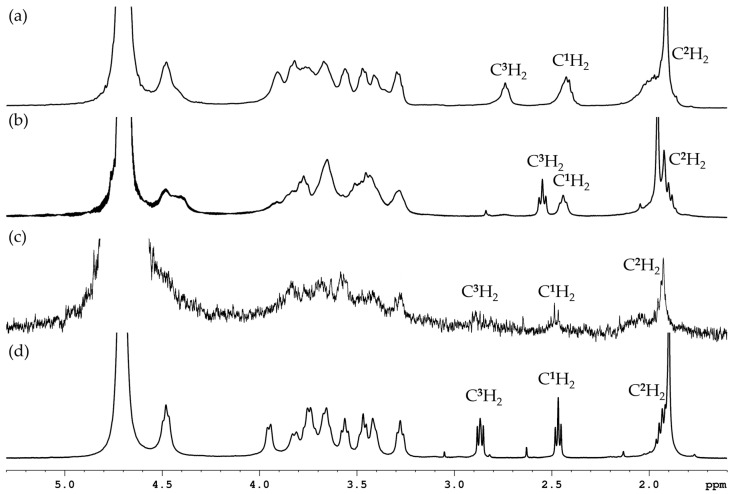
^1^H NMR spectra of compounds in D_2_O: (**a**) HA–S–S–HA (**1**); (**b**) HA–SH (**2**); (**c**) result of the reaction of **2** with HAuCl_4_ at a ratio of [**2**]:[HAuCl_4_] = 1:0.20; and (**d**) result of the reaction of **2** with HAuCl_4_ at a ratio of [**2**]:[HAuCl_4_] = 1:1.

In the ^1^H NMR spectrum of compound **1**, there is a broadened signal in the region δ 2.74 ppm, which corresponds to the protons of the methylene group at the –S–S– bond (–C**^3^**H_2_–S–), and in the region δ_H_ 2.43 ppm related to the protons of the methylene group at C=O (Figure 5a). ^1^H NMR spectrum of compound **2** exhibits a signal at δ_H_ 2.55 ppm, corresponding to the protons of the methylene group at the –SH bond (–C^3^H_2_–SH), and δ_H_ 2.44 ppm, which relates to the methylene group at C=O (Figure 5b), while the resonance lines of hydrogen atoms at the C2 atom in both cases overlapped with the N-acetylmethyl protons of HA. The spectral data of compound **2** were consistent with the literature [57].

As indicated by the ^1^H and ^13^C NMR spectra of the reaction mixture obtained after the reaction of **2** with HAuCl_4_, the thiol group undergoes significant changes, the result of which is apparently the formation of sulfonic acid. This is evidenced by the appearance of a new set of signals of –(CH_2_)_3_‒ fragments at δ_H_ 2.02 ppm, 2.43 ppm, and 2.89 ppm in the ^1^H NMR spectra shifted to the downfield relative to the corresponding signals of compound **2** (Figure 5c). The intensity of these signals raised as the amount of HAuCl_4_ introduced increased (Figure 5d). The multiplicity of the downfield signal of the protons of the CH_2_ fragment at δ_H_ 2.89 corresponded to the pattern AA’BB’ of the CH_2_-CH_2_ group (J_AB_ = 6.5, J_AB’_ = 9.0 Hz) with a predominance of the gauche conformation over the anti-conformation [58], which may be a consequence of the appearance of a symmetrical sulfonate group, limiting the conformational mobility of the hydrocarbon chain. The appearance of the sulfonate fragment is also indicated by the shift in the signal of the carbon atom bonded to the sulfur atom from δ_C_ 23.03 ppm in compound **2** to δ_C_ 49.90 ppm (Figure S13) [59,60].

Thus, it can be assumed that along with the formation of AuNPs decorated with -S-HA, oxidation of the thiol group to sulfonic acid takes place, as represented in Scheme 1. The process could occur with the participation of H_2_O_2_, the formation of which is possible as a result of the spontaneous reduction of Au^3+^ ions in HAuCl_4_ solutions to Au^+^ ions and Au nanoparticles [47], or with O_2_. The interaction of HA–SH with Au^3+^ and hydrogen peroxide or oxygen can lead to cross-linking of the SH bonds with their transformation into HA–S–S–HA [61,62,63] and/or to the formation of sulfonic acid (Scheme 1) [64,65,66].

#### 2.2.5. ζ-Potential Measurements

To assess the electrostatic stability of colloidal systems, the ζ-potential was measured. The samples were analyzed in an aqueous medium at pH ~ 5–6 and room temperature. The obtained negative values of ζ-potential, presented in Table 3, indicate that AuNPs were coated with anionic HA [24]. AuNPs obtained at HA-to-HAuCl_4_ ratios of 1:0.25 and 1:0.225 showed a ζ-potential of –27.6 and –28.6 mV, respectively, whereas at lower Au concentrations of 1:0.20 and 1:0.175, a significant increase in negative charge to −41.0 and −41.5 mV, respectively, was observed, which indicates an increase in system stability [67,68]. The resulting sulfonic acid derivative of HA can also participate in the stabilization of gold nanoparticles.

These data indicate that the use of different concentrations of HAuCl_4_ affects the surface properties of the obtained AuNPs. More negative values of ζ-potential with decreasing amounts of HAuCl_4_ may indicate more effective coating or stabilization of the nanoparticles by hyaluronic acid, which leads to an increase density of negatively charged functional groups on the surface. This contributes to an increase in electrostatic repulsion between particles, which in turn improves the stability of the system. On the other hand, at higher HAuCl_4_ content (1:0.25 and 1:0.225), less negative values of ζ-potential are observed, which may be related to the partial neutralizing effect of gold ions or to a decrease in the availability of negatively charged HA groups on the nanoparticle surface. This can lead to a reduction in electrostatic stabilization and an increase in the probability of aggregation.

Thus, changing the ratio of thiol-modified HA to HAuCl_4_ has a significant effect on the charge characteristics of the nanoparticle surface and, consequently, on their colloidal stability. Optimization of this parameter is a key factor for obtaining stable and functional nanomaterials based on hyaluronic acid and gold.

## 3. Materials and Methods

### 3.1. General Information

The following reagents were used for the synthesis: the low-molecular-weight (LMW) HA (<0.1 MDa), 4,4′-Dithiodibutyric acid (95%, Sigma-Aldrich Chemie GmbH, Steinheim, Germany), 1-methyl-3-(3-dimethylaminopropyl)carbodiimide hydrochloride (EDC*HCl, 98%, abcr GmbH, Karlsruhe, Germany), Cleland’s Reagent (DTT, 98%, abcr GmbH, Karlsruhe, Germany). 4,4′-Dithiodibutyric acid dihydrazide (DTDBA) was obtained by the method [69], and the SH derivative of HA (**2**) was obtained according to a known procedure [57].

Spectroscopic studies were carried out by means of ^1^H and ^13^C on an AVANCE-500 spectrometer (Bruker, Rheinstetten, Germany; operating frequency 500.17 MHz (^1^H) and 125.78 MHz (^13^C)). D_2_O and CDCl_3_ were used as internal standards and solvents. Samples were prepared in a standard ampoule with a diameter of 5 mm. The chemical shifts of hydrogen atoms are given in the scale δ (ppm) relative to tetramethylsilane (TMS). One- and two-dimensional NMR spectra (COSY ^1^H–^1^H, HSQC, HMBC, NOESY, and DOSY) were recorded using standard Bruker pulse sequences.

### 3.2. Preparation of HA-AuNPs

AuNPs were synthesized by the reaction of HA-SH (**2**) with HAuCl_4_. Into an aqueous solution of HA-SH (**2**) with a concentration of 4.6 mg/mL under intensive stirring, an aqueous solution of HAuCl_4_ (at a concentration of 10 mg/mL) was added at mass ratios of HA to HAuCl_4_ from 1:1 to 1:0.1, respectively. The reaction mixtures were heated and stirred at 90 °C for 2 h. Then, they were cooled to room temperature and stirred at room temperature for 12 h.

### 3.3. Characterization of AuNPs

The microstructure of the samples was studied using scanning electron microscopy (SEM) and bright-field scanning transmission electron microscopy (BF-STEM) on a Hitachi Regulus 8220 electron microscope (Japan). Before measurements the samples were deposited on the 3 mm carbon-coated copper grids from water suspension. Images were acquired in transmitted electron mode at 30 kV accelerating voltage.

X-ray photoelectron spectroscopy (XPS) spectra were obtained using a JEOL JPS 9010MX spectrometer (JEOL, Tokyo, Japan) equipped with an X-ray source (Mg Kα). The pressure in the analytical chamber during spectrum acquisition was less than 7·10^−8^ Pa. The samples of AuNP solutions were deposited on titanium plates (Grade 4) and then dried from the solvent. Spectra were collected from 0 to 1100 eV with a pass energy of 50 eV and a step size of 0.5 eV. Binding energies (BEs) were corrected by adjusting the position of the C1s peak to 284.8 eV. The JEOL SpecSurf software was used to determine peak areas, calculate elemental composition from peaks, and fit peaks to high-resolution spectra. Deconvolution of the spectra was performed using the Voigt function with the JEOL SpecSurf v. 1.9.0 software.

Optical properties of AuNPs solutions were determined by scanning all samples at 250 nm to 800 nm using the UV–Vis spectrophotometer UV-1800 (Shimadzu, Tokio, Japan).

The particle size distribution was studied by the means of Photon Cross-correlation Spectroscopy (PCCS) implemented in the NanoPhox (Sympatec, Clausthal-Zellerfeld, Germany). Particle size analysis was performed using the PAQXOS 4.2 program. Each sample was measured three times at 25 °C. Nanosphere™ Size Standards (ThermoFisher Scientific, Waltham, MA, USA) with particle sizes 23 ± 2, 100 and 510 ± 7 nm were examined prior to analysis to verify the accuracy.

The SMH ζ-potential was measured by the optical heterodyning technique, using a Zetasizer Nano ZS (Malvern Instruments Ltd., Malvern, UK). In this technique, laser radiation scattering is used to measure the velocity of charged particles in an electric field applied to a cell through a pair of electrodes.

## 4. Conclusions

In this study, we have demonstrated the synthesis of gold nanoparticles using thiolated hyaluronic acid (HA–SH) as a synthetic and stabilization agent. A series of gold nanoparticles (**HA-AuNP1-4**) were synthesized by varying the mass ratio of HA–SH to HAuCl_4_ at the same initial concentration of the polysaccharide. The concentration of the gold precursor was found to be a critical parameter, directly influencing the size, morphology, and optical properties of the resulting AuNPs, as evidenced by UV-Vis, PCCS, SEM, and STEM analyses.

The investigation revealed that the reaction involves not only the reduction of Au^3+^ to form nanoparticles but also a concurrent chemical transformation of the thiol group. Comprehensive NMR and XPS analyses confirmed the oxidation of the thiol (–SH) groups to sulfonic acid (–SO_3_H), suggesting a complex redox process alongside nanoparticle formation. Nevertheless, XPS analysis confirmed the metallic nature of the gold core (Au^0^) and the presence of a Au–S bond, proving the direct involvement of the thiolated polymer in capping the nanoparticles.

The synthesized AuNPs were effectively stabilized by the anionic hyaluronic acid shell, as indicated by negative zeta potential values and SEM images.

In conclusion, thiolated hyaluronic acid serves as an effective platform for the one-pot synthesis and stabilization of gold nanoparticles with controllable characteristics. The unveiled oxidation pathway adds a new dimension to the understanding of the synthesis mechanism. The resulting HA-AuNPs, with their tunable properties, represent promising candidates for further development in targeted drug delivery, theranostics, and other biomedical applications.

## Figures and Tables

**Table 1:** UV-Vis absorption maxima (λmax, nm) for **HA-AuNP1-4**.

Sample	λmax (1 Day), nm	λmax (30 Days), nm
**HA-AuNP1**	557	-
**HA-AuNP2**	563	-
**HA-AuNP3**	555	559
**HA-AuNP4**	569	-

**Table 2:** Fitting parameters of the Au 4f spectrum for HAuCl_4_ and **HA-AuNP3**.

Sample	Au	Binding Energy (eV) 4f_7/2_	Binding Energy (eV) 4f_5/2_	Rel. Area (%)
HAuCl_4_	Au^3+^	86.9	90.6	47.88 ± 1.06
	Au^1+^	85.3	88.9	35.62 ± 0.79
	Au^0^	84.6	88.3	16.48 ± 0.36
**HA-AuNP3**	Au^3+^	-	-	-
	Au^1+^	84.6	88.3	40.79 ± 1.23
	Au^0^	84.1	87.8	59.21 ± 1.31

**Table 3:** Values of ζ-potentials of samples **HA-AuNP1-4**.

	HA-AuNP1	HA-AuNP2	HA-AuNP3	HA-AuNP4
Zeta-potential (mV)	−27.6 ± 3.4	−28.6 ± 1.7	−41.0 ± 4.0	−41.5 ± 1.3

## Data Availability

The datasets generated during and/or analyzed during the current study are available from the corresponding author on reasonable request.

## Supplementary Materials

The following supporting information can be downloaded at https://www.mdpi.com/article/10.3390/ijms262110532/s1.
