# Supplementary material for: Au Nanoparticle Synthesis in the Presence of Thiolated Hyaluronic Acid
Source: Int J Mol Sci. 2025 Oct 29;26(21):10532. doi: 10.3390/ijms262110532 (PMC12607323; doi:10.3390/ijms262110532)
Supplement: Supplementary file 1 [file ijms-26-10532-s001.zip › ijms-3941371-supplementary.pdf]

Au Nanoparticle Synthesis in the Presence of Thiolated Hyaluronic Acid

Lyudmila V. Parfenova,[1]* Eliza I. Alibaeva[1], Guzel U. Gil'fanova[1], Zulfiya R. Galimshina[1], Ekaterina S. Mescheryakova,[1] Leonard M. Khalilov,[1] Semen N. Sergeev,[2] Nikita V. Penkov,[3] Challapalli Subrahmanyam[4]

[1] Institute of Petrochemistry and Catalysis, Ufa Federal Research Center, Russian Academy of Sciences, Prosp. Oktyabrya, 141, 450075 Ufa, Russia

[2] Ufa University of Science and Technology, 12 Karl Marx Street, 450008 Ufa, Russia

[3] Institute of Cell Biophysics of the Russian Academy of Sciences, Federal Research Center "Pushchino Scientific Center for Biological Research of the Russian Academy of Sciences", Institutskaya 3, 142290 Pushchino, Russia

[4]Department of Chemistry, Indian Institute of Technology Hyderabad, Sangareddy, Kandi, Telangana 502285, India

* Author to whom correspondence should be addressed: luda_parfenova@mail.ru

Supporting Information

Figure S1. PCCS of **HA-AuNP1** obtained in mass ratio system [**2**]:[$HAuCl_4$]**=**1:0.25 3

Figure S2. PCCS of **HA-AuNP2** obtained in mass ratio system [**2**]:[$HAuCl_4$]**=**1:0.25 3

Figure S3. PCCS of **HA-AuNP3** obtained in mass ratio system [**2**]:[$HAuCl_4$]**=**1:0.225 (1 day) 4

Figure S4. PCCS of HA-AuNP3 obtained in mass ratio system [**2**]:[$HAuCl_4$]=1:0.225 (30 days) 4

Figure S5. BF-STEM and SEM images of **HA-AuNP1**. 5

Figure S6. BF-STEM and SEM images of **HA-AuNP2**. 7

Figure S7. BF-STEM and SEM images of **HA-AuNP3**. 8

Figure S8. NMR ^{1}H spectra of compound **2**..... 9

Figure S9. NMR ^{13}C spectra of compound **2** 9

Figure S10. NMR 2D COSY HH spectra of compound **2**..... 10

Figure S11. NMR 2D HSQC spectra of compound **2**..... 10

Figure S12. NMR ^{1}H spectra of the reaction mixture of **2** with $HAuCl_4$ at a ratio of [**2**]:[$HAuCl_4$]= 1:1..... 11

Figure S13. NMR ^{13}C spectra of the reaction mixture of **2** with $HAuCl_4$ at a ratio of [**2**]:[$HAuCl_4$]= 1:1..... 11

Figure S14. NMR 2D COSY HH spectra of the reaction mixture of **2** with $HAuCl_4$ at a ratio of [**2**]:[$HAuCl_4$]= 1:1. 12

Figure S15. NMR 2D HSQC spectra of the reaction mixture of **2** with $HAuCl_4$ at a ratio of [**2**]:[$HAuCl_4$]= 1:1 12

Table S1. Positions and FWHM of the XPS characteristic peaks of the $HAuCl_4$ and **HA-AuNP3**. 13

Figure S1. PCCS of **HA-AuNP1** obtained in mass ratio system [**2**]:[$HAuCl_4$]=1:0.25

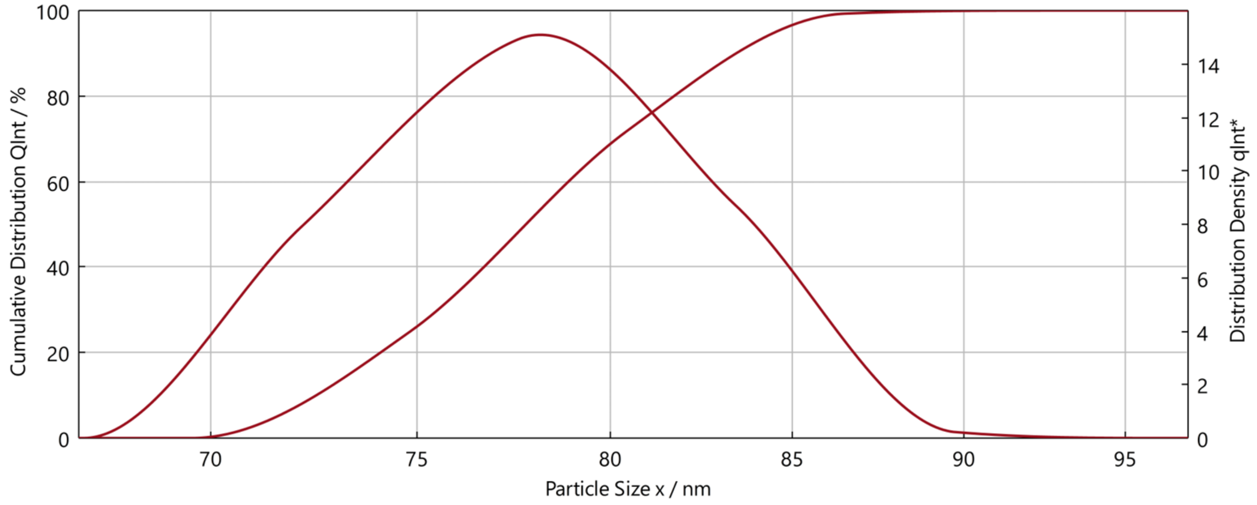

Figure S2. PCCS of **HA-AuNP2** obtained in mass ratio system [**2**]:[$HAuCl_4$]=1:0.25

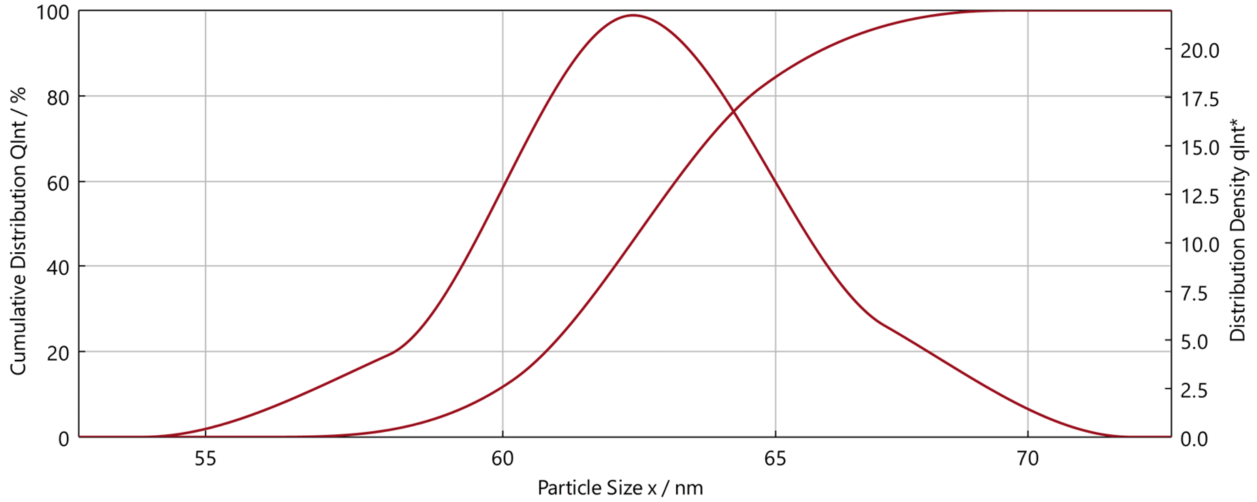

Figure S3. PCCS of **HA-AuNP3** obtained in mass ratio system [**2**]:[$HAuCl_4$]**=**1:0.225 (1 day)

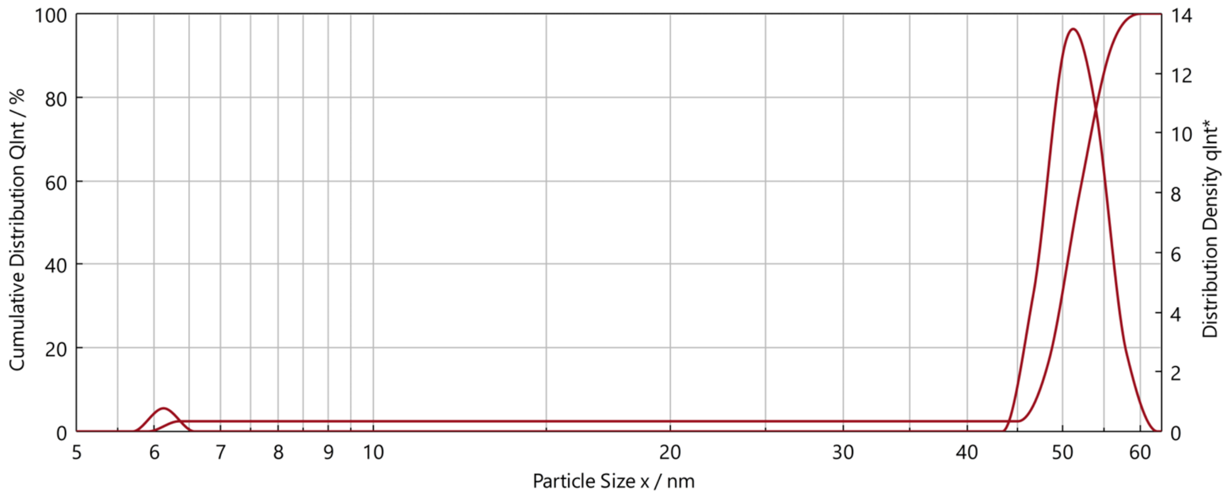

Figure S4. PCCS of HA-AuNP3 obtained in mass ratio system [**2**]:[$HAuCl_4$]=1:0.225 (30 days)

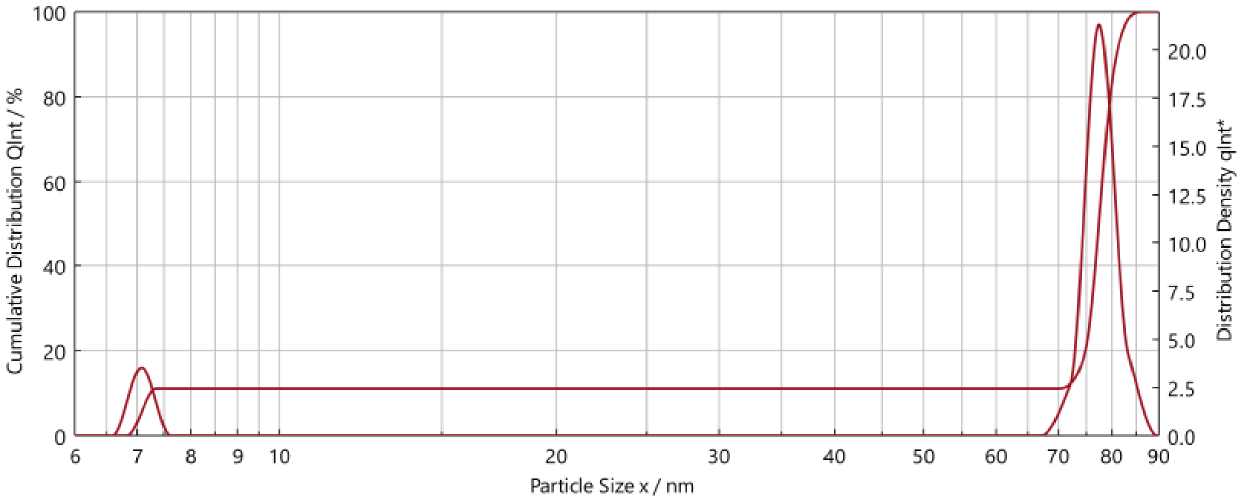

Figure S5. BF-STEM and SEM images of **HA-AuNP1**.

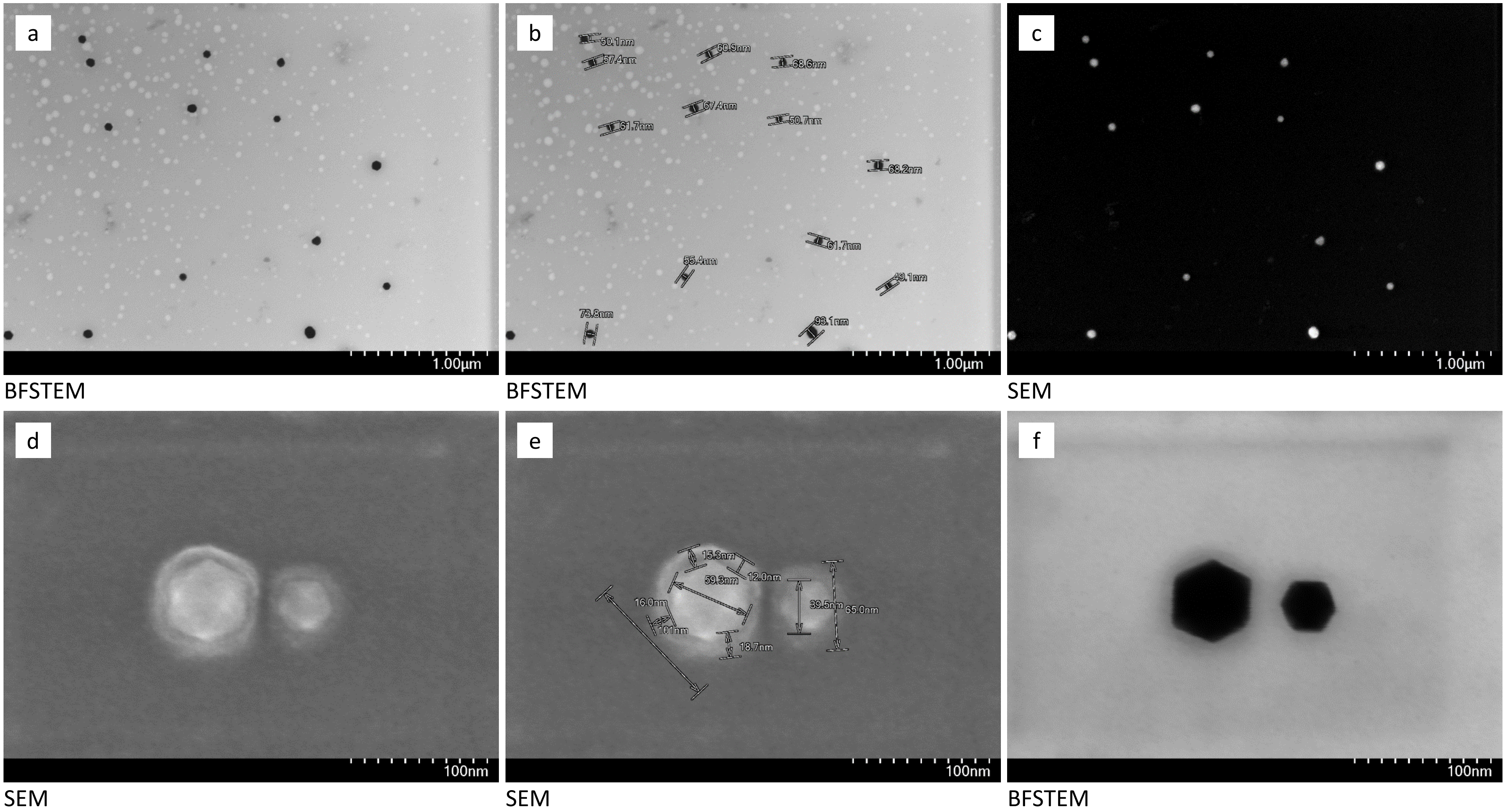

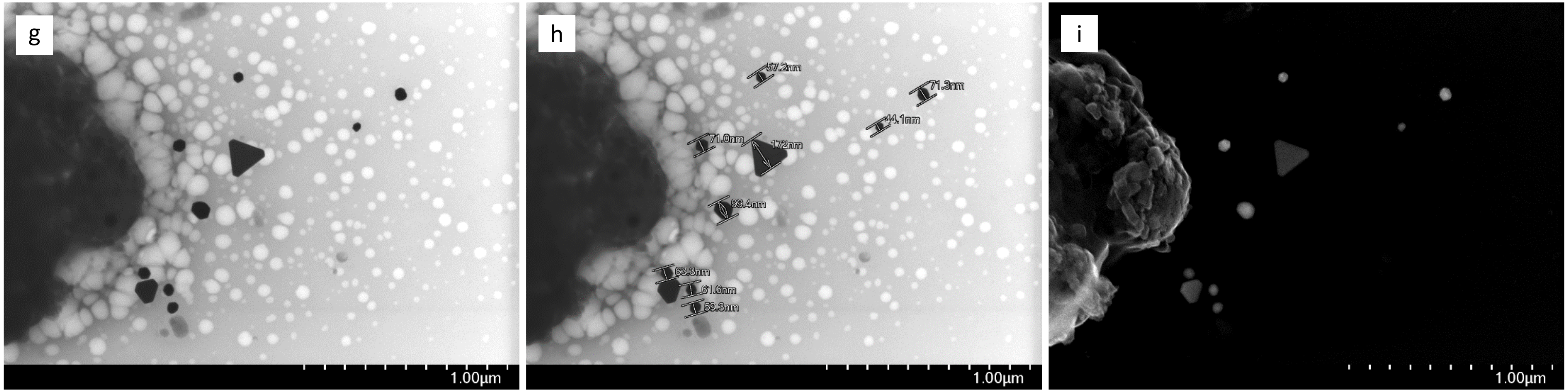

BFSTEM BFSTEM SEM

Figure S6. BF-STEM and SEM images of **HA-AuNP2**.

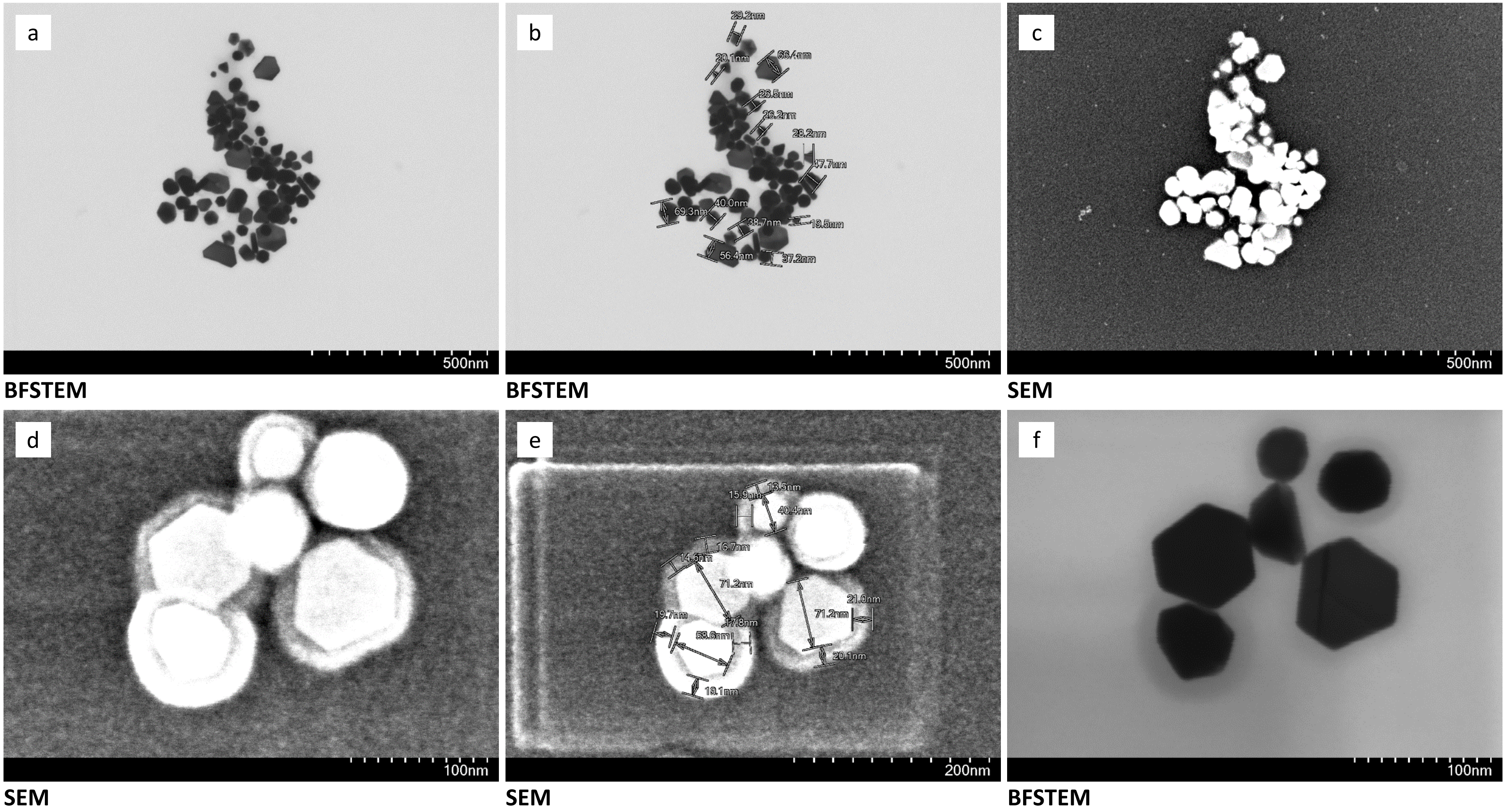

Figure S7. BF-STEM and SEM images of **HA-AuNP3**.

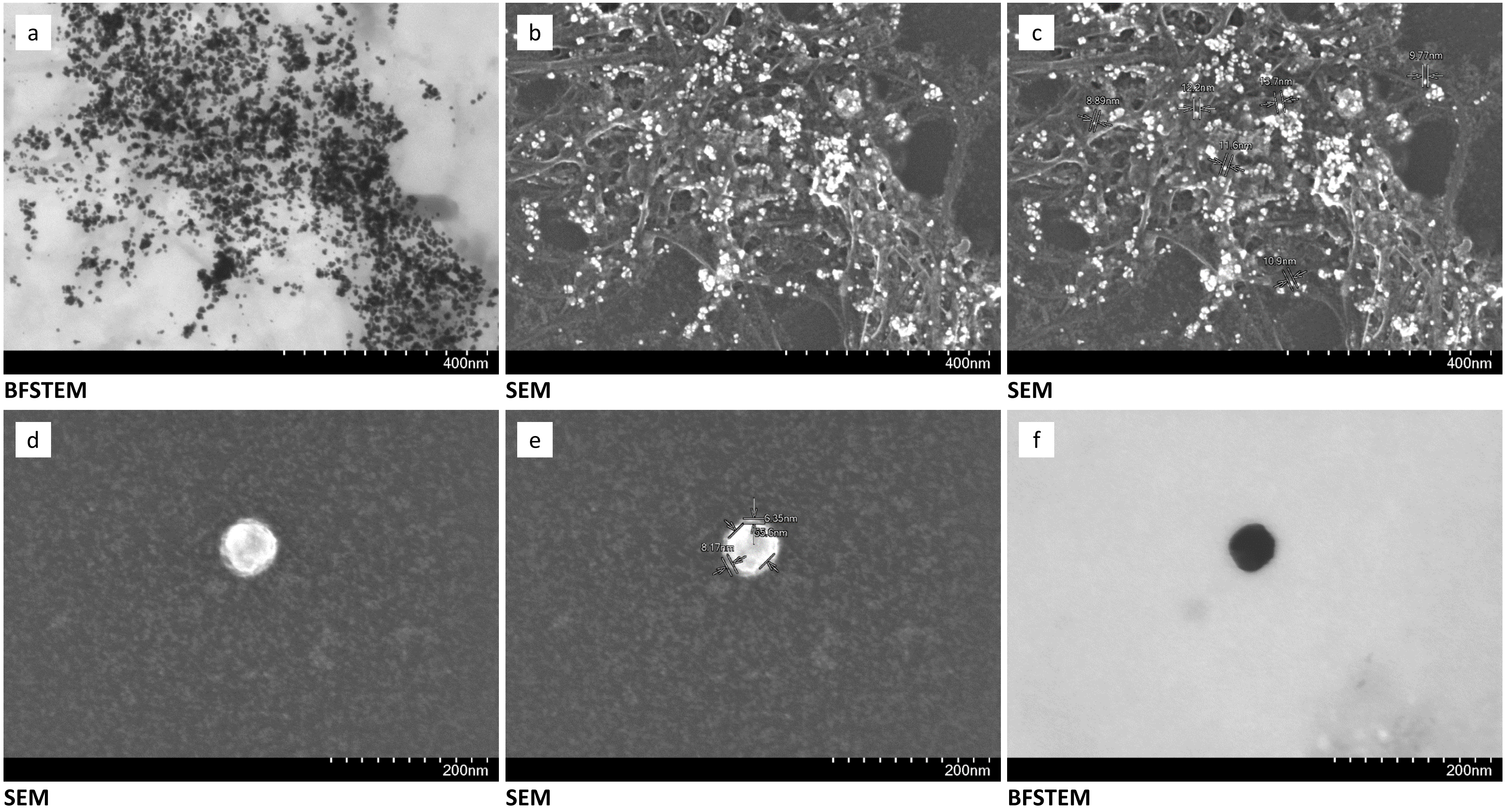

Figure S8. NMR ^{1}H spectra of compound **2**

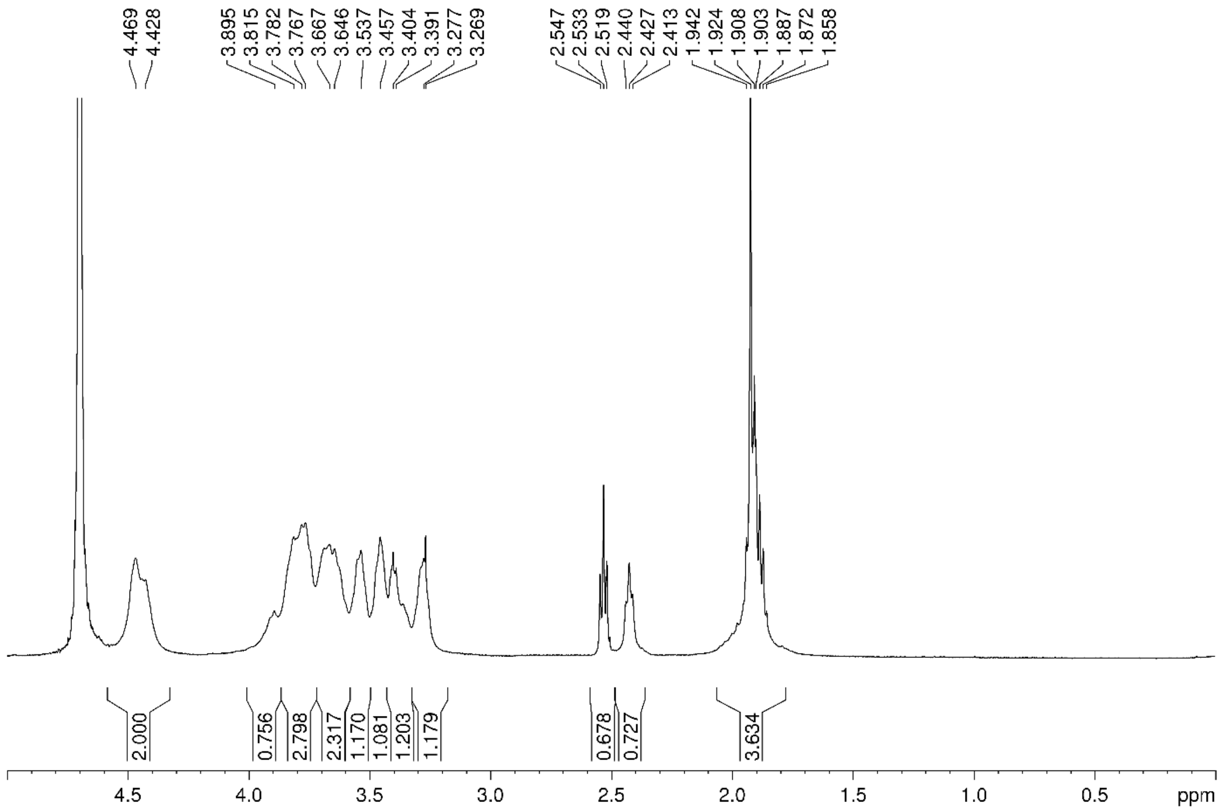

Figure S9. NMR ^{13}C spectra of compound **2**

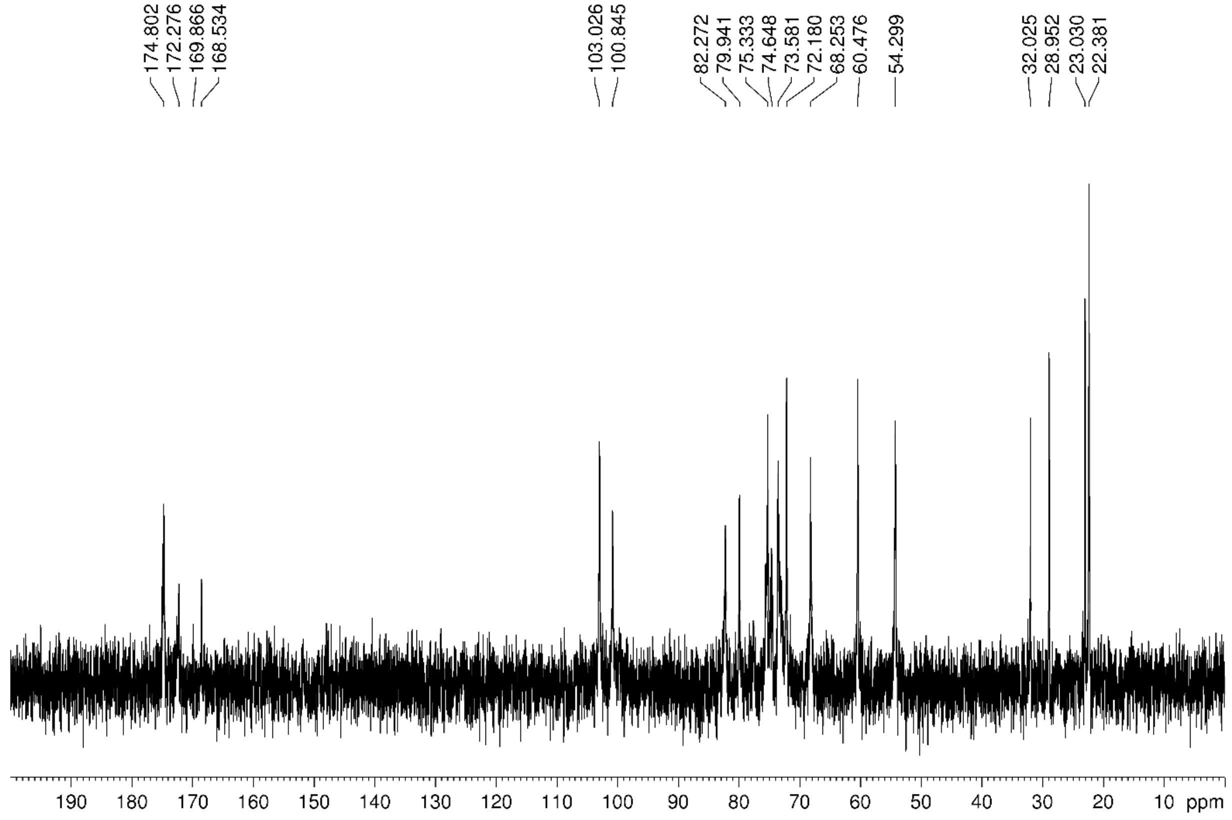

Figure S10. NMR 2D COSY HH spectra of compound **2**

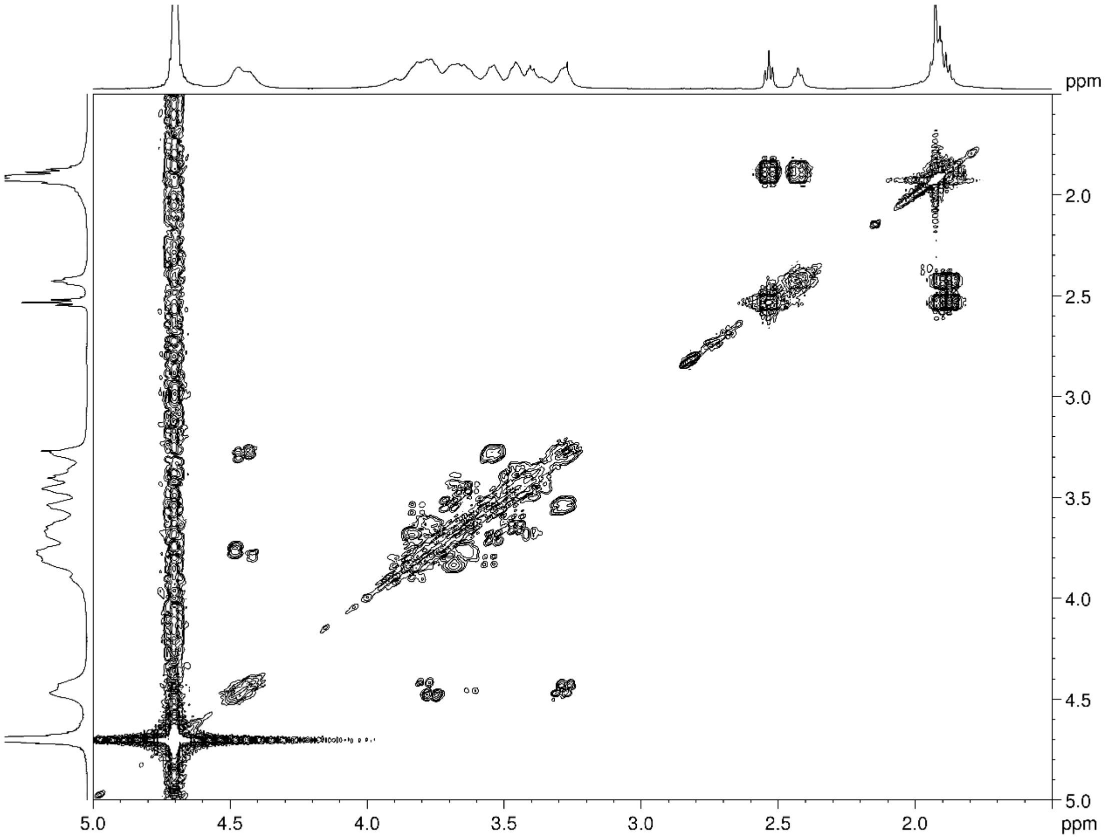

Figure S11. NMR 2D HSQC spectra of compound **2**

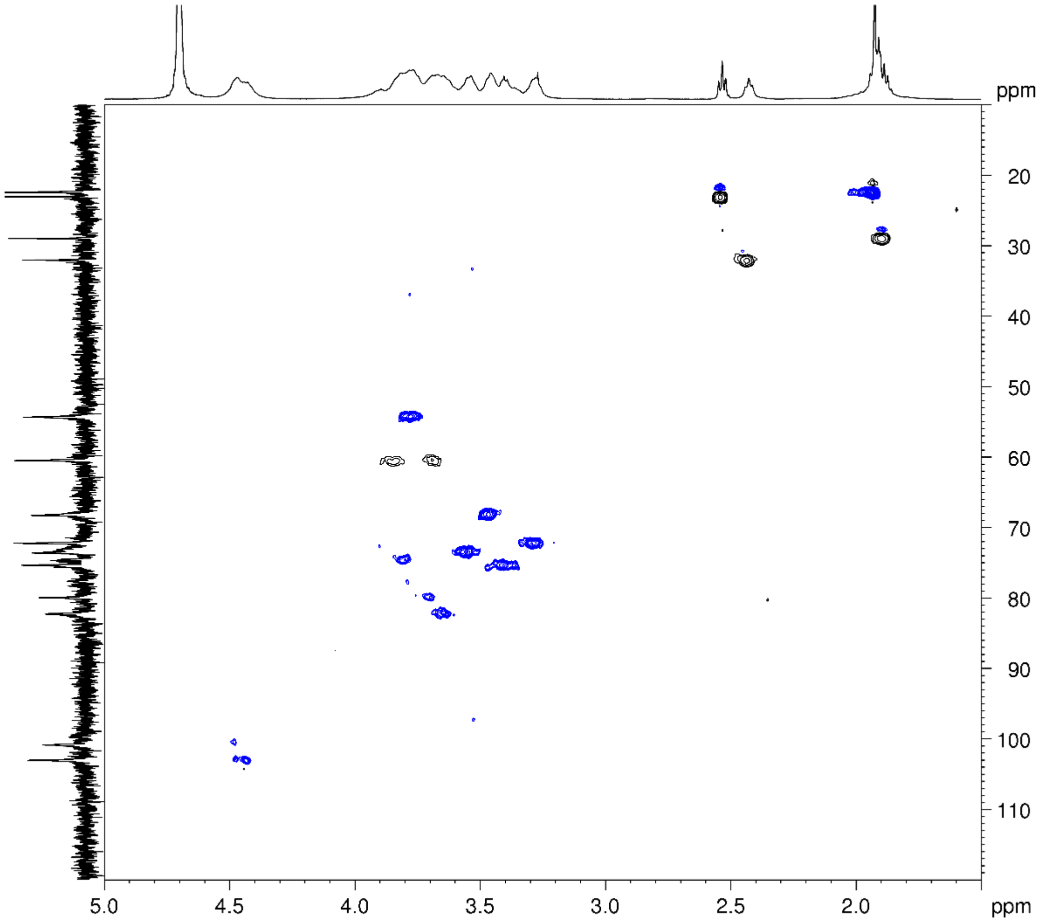

Figure S12. NMR ^{1}H spectra of the reaction mixture of **2** with $HAuCl_4$ at a ratio of [**2**]:[$HAuCl_4$]= 1:1.

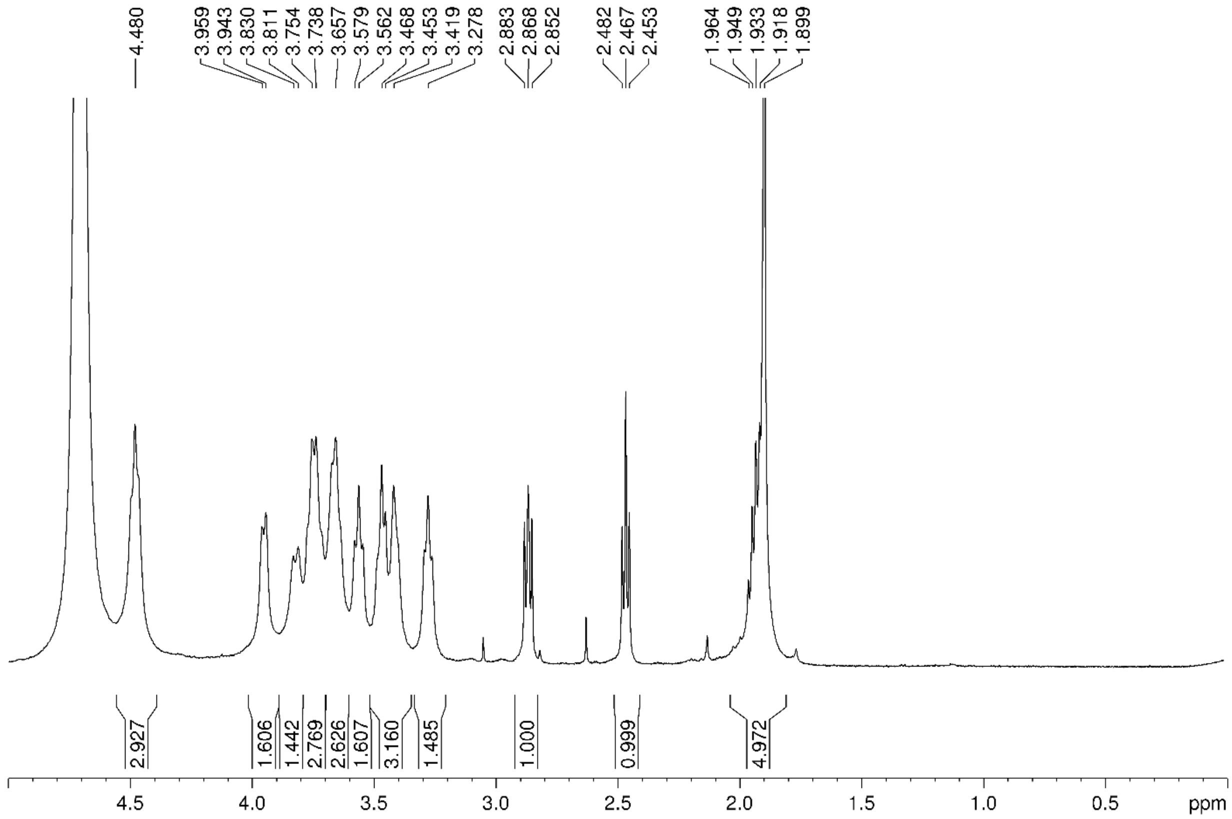

Figure S13. NMR ^{13}C spectra of the reaction mixture of **2** with $HAuCl_4$ at a ratio of [**2**]:[$HAuCl_4$]= 1:1

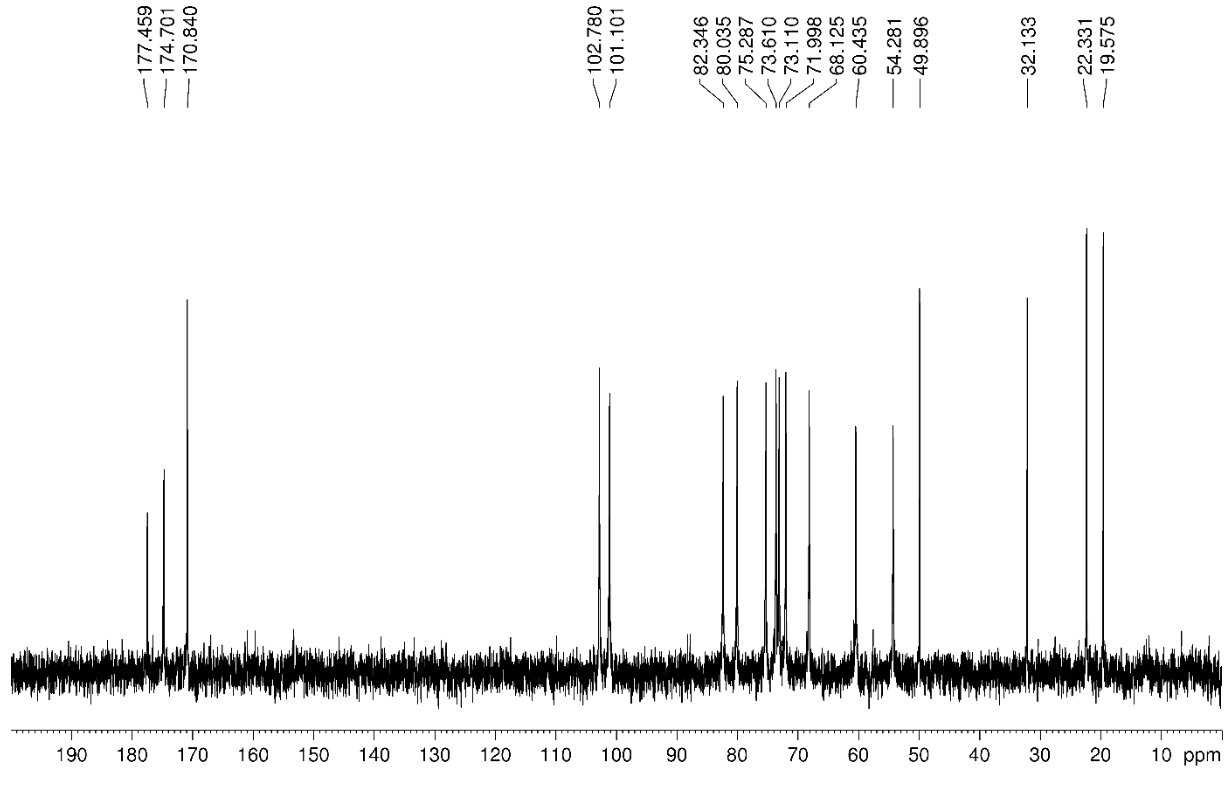

Figure S14. NMR 2D COSY HH spectra of the reaction mixture of **2** with $HAuCl_4$ at a ratio of [**2**]:[$HAuCl_4$]= 1:1.

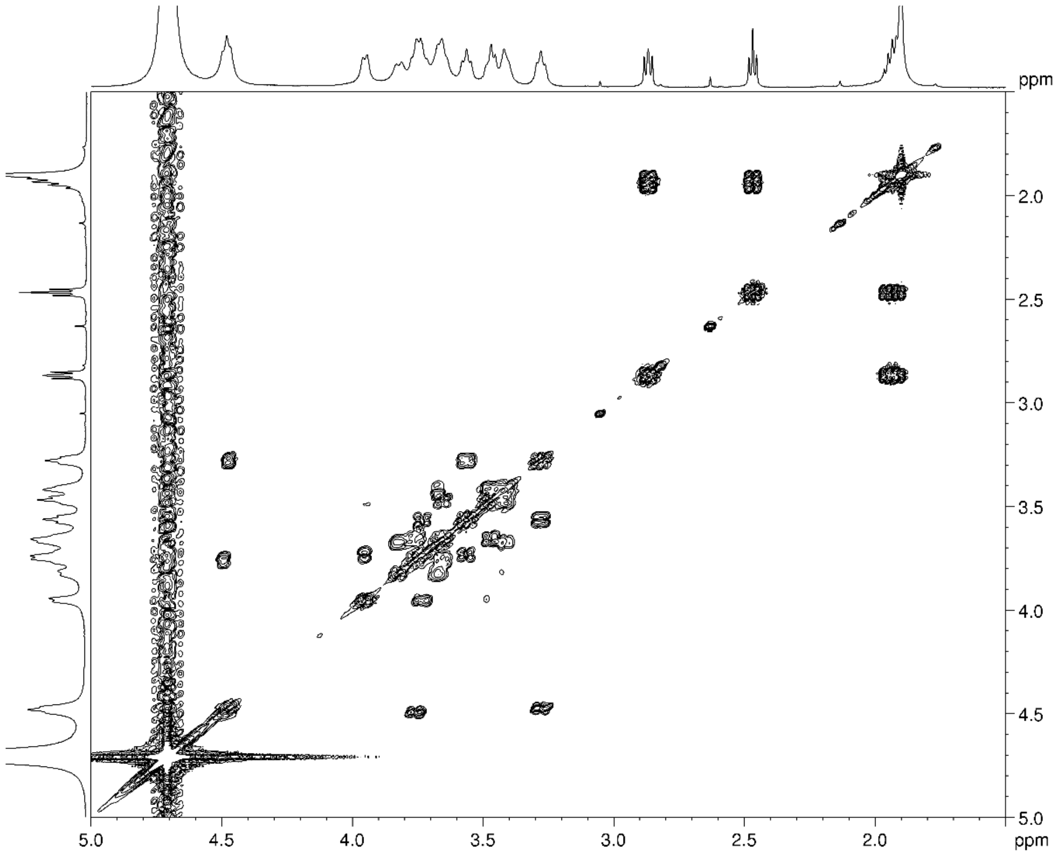

Figure S15. NMR 2D HSQC spectra of the reaction mixture of **2** with $HAuCl_4$ at a ratio of [**2**]:[$HAuCl_4$]= 1:1.

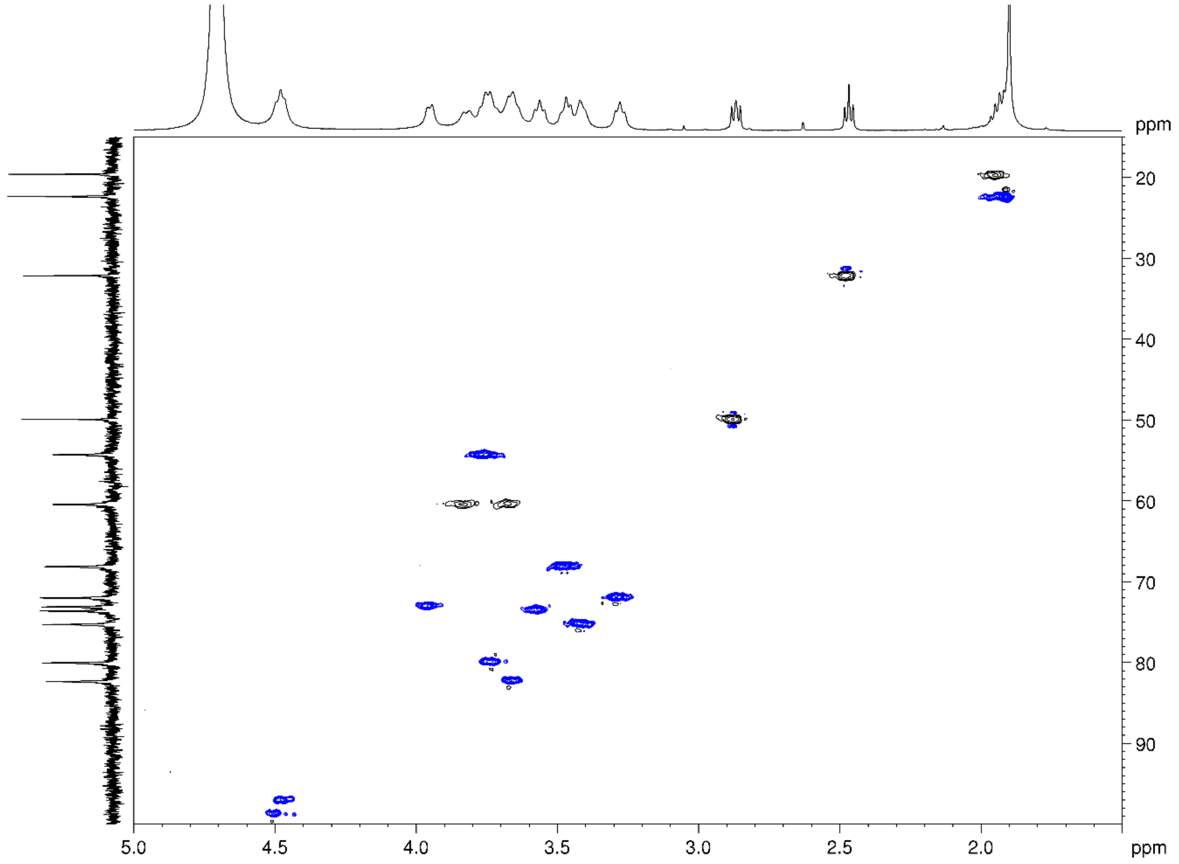

Table S1. Positions and FWHM of the XPS characteristic peaks of the $HAuCl_4$ and **HA-AuNP3**.

Sample code	Peak	Position (eV)	FWHM (eV)	Position (eV)	FWHM (eV)	Position (eV)	FWHM (eV)
$HAuCl_4$	Au4f 5/2	90.588	0.766	88.912	0.482	88.296	0.413
	Au4f 7/2	86.912	0.766	85.246	0.482	84.635	0.413
	C1s	284.825	0.749	-	-	-	-
	O1s	533.216	2.487	-	-	-	-
**HA-AuNP3**	Au4f 5/2	88.299	0.547	87.769	0.641	-	-
	Au4f 7/2	84.627	0.547	84.084	0.641	-	-
	C1s	288.174 (8.28%)	0.754	286.460 (26.84%)	0.756	284.789 (64.88%)	0.649
	O1s	533.117 (46.98%)	0.815	532.206 (25.43%)	0.688	530.416 (27.59%)	0.895
	S2p 1/2	169.548 (15.94%)	0.716	164.314 (17.40%)	0.764	-	-
	S2p 3/2	168.371 (31.85%)	0.716	163.136 (34.81%)	0.764	-	-
